# Two-stage classification strategy for breast cancer diagnosis using ultrasound-guided diffuse optical tomography and deep learning

**DOI:** 10.1117/1.JBO.28.8.086002

**Published:** 2023-08-26

**Authors:** Menghao Zhang, Shuying Li, Minghao Xue, Quing Zhu

**Affiliations:** a Washington University in St. Louis, Department of Electrical and Systems Engineering, St. Louis, Missouri, United States; b Washington University in St. Louis, Department of Biomedical Engineering, St. Louis, Missouri, United States; c Washington University School of Medicine, Department of Radiology, St. Louis, Missouri, United States

**Keywords:** diffuse optical tomography, ultrasound, breast cancer diagnosis, deep learning, classifier

## Abstract

**Significance:**

Ultrasound (US)-guided diffuse optical tomography (DOT) has demonstrated great potential for breast cancer diagnosis in which real-time or near real-time diagnosis with high accuracy is desired.

**Aim:**

We aim to use US-guided DOT to achieve an automated, fast, and accurate classification of breast lesions.

**Approach:**

We propose a two-stage classification strategy with deep learning. In the first stage, US images and histograms created from DOT perturbation measurements are combined to predict benign lesions. Then the non-benign suspicious lesions are passed through to the second stage, which combine US image features, DOT histogram features, and 3D DOT reconstructed images for final diagnosis.

**Results:**

The first stage alone identified 73.0% of benign cases without image reconstruction. In distinguishing between benign and malignant breast lesions in patient data, the two-stage classification approach achieved an area under the receiver operating characteristic curve of 0.946, outperforming the diagnoses of all single-modality models and of a single-stage classification model that combines all US images, DOT histogram, and imaging features.

**Conclusions:**

The proposed two-stage classification strategy achieves better classification accuracy than single-modality-only models and a single-stage classification model that combines all features. It can potentially distinguish breast cancers from benign lesions in near real-time.

## 1 Introduction

Breast cancer is the most common cancer among women in the United States, with around 2.9 million estimated new cases in 2022.^1^ X-ray mammography is the major modality for both screening and diagnosis;^2^^,^^3^ however, it has limited sensitivity when used in dense breasts.^4^ For average-risk patients with dense breasts, breast ultrasound (US) has been widely adopted as both a diagnostic tool and supplementary screening tool.^5^^,^^6^ Magnetic resonance imaging is also a supplementary tool for screening dense breasts of high-risk women,^7^ but its high cost makes it accessible to only a small patient population. Despite improvements in imaging tools, a low positive predictive value for biopsy recommendation has remained a problem. In 2017, the Breast Cancer Surveillance Consortium reported only a 27.5% positive predictive value for biopsy recommendation.^2^

In the last two decades, diffuse optical tomography (DOT), which utilizes diffused near-infrared (NIR) light to map tissue optical properties, has been widely explored for non-invasive breast cancer diagnosis.^8^^–^^22^ US-guided DOT, utilizing co-registered US images to provide lesion size and depth information for improved DOT reconstruction accuracy, has shown success in breast cancer diagnosis and treatment monitoring.^23^^–^^28^ A recent study reported that the adjunctive use of US-guided DOT can reduce benign biopsies by 23.5%.^29^

Recently, machine learning (ML) has been applied to DOT in bulk tissue optical property estimation,^30^^,^^31^ image reconstruction,^32^^–^^39^ and breast cancer diagnosis.^40^^–^^43^ Using simulated breast lesions, Di Sciacca et al.^42^ applied logistic regression, support vector machines (SVMs), and a fully connected network to reconstruct optical properties and achieved a best accuracy of 78%. Xu et al.^43^ applied a convolutional neural network (CNN) to 1260 2D optical tomographic images sliced from 3D images collected from 63 women with dense breasts and achieved a 0.95 area under the receiver operating characteristic (ROC) curve (AUC). Inspired by recent multi-model fusion techniques in medical imaging,^44^^–^^46^ Zhang et al. combined US features extracted by a modified VGG-11 network with DOT images to form a new neural network for classification, and it achieved an AUC of 0.931 in distinguishing between benign and malignant breast lesions.^40^ However, DOT’s relatively slow data processing and image reconstruction speeds have hindered real-time diagnosis. To address this problem, our group has developed a two-stage strategy.^41^ The first stage uses breast imaging reporting and data system (BI-RADS) readings from radiologists together with features from DOT frequency domain perturbation measurement data represented in two-dimensional histograms (DOT histograms). These data are combined by a random forest classifier to screen out ∼60% of benign lesions without the need of reconstruction. The second stage then utilizes reconstructed DOT images and an SVM classifier to improve the overall diagnostic accuracy. However, this strategy relies on radiologists’ readings, which can be unavailable or have limited accuracy in remote or low-resource settings. Additionally, it relies on limited hand-crafted features from DOT measurement data and DOT images, which may not capture all important features.

In this study, we further improve the two-stage classification strategy with deep learning and we automate it to achieve faster and more accurate classification for breast lesions without BI-RADS readings or hand-crafted features. In the first stage, we train two CNN-based classifiers using US images and DOT histograms. The predictions of the CNNs are averaged to single out benign lesions. The rest of the non-benign suspicious lesions pass through the second stage, which combines US image features, DOT histograms, and DOT reconstructed images for diagnosis of this group. This two-stage classification strategy combines the diagnoses of first and second stage classifiers and has achieved the excellent diagnostic performance by filtering out 70% of the benign lesions in near real-time without DOT image reconstruction.

## 2 Methods

### 2.1 Data

A total of 254 patients (169 with benign lesions and 85 with malignant lesions) were studied to evaluate the proposed diagnostic strategy. In the benign group, the maximum dimensions of the lesions, measured by US, ranged from 0.31 to 5.30 cm, with a mean of 1.48 cm, and the depths ranged from 0.65 to 3.20 cm, with a mean of 1.50 cm. In the malignant group, the maximum dimensions of the lesions ranged from 0.43 to 5.52 cm, with a mean of 2.05 cm, and the depths ranged from 0.70 to 3.00 cm, with a mean of 1.53 cm. DOT patient data and co-registered US images were acquired with our US-guided DOT systems at four wavelengths in the NIR range.^24^^,^^47^ The clinical study was approved by the local Institutional Review Boards and was compliant with the Health Insurance Portability and Accountability Act. Informed consent was obtained from each patient, and data from patients were de-identified. Because of our limited dataset, the results could be influenced by the training-testing splits. Thus, we chose to employ a bootstrapping approach to evaluate the performance of our models by shuffling the dataset 50 times, providing a more robust model performance than using a small testing dataset. To use a balanced dataset, in each of the 50 runs, 120 lesions (60 benign and 60 malignant) were selected as the training set and the other 50 (25 benign and 25 malignant) were used as the testing set. The hyperparameters were tuned based on the averaged performance of the 50 runs. In addition, using the finite element method (FEM) and Monte Carlo (MC) simulations, we generated a total of 880 sets of DOT measurements to pretrain the DOT models before fine-tuning the models with patient data. For FEM simulation, we used COMSOL software to generate the forward measurements. One hemisphere was used to simulate breast tissue, and the other layer was used to simulate the chest wall.^37^ The VICTRE digital breast phantoms were incorporated with Monte Carlo simulation to simulate heterogeneous breast tissue.^48^^,^^49^ The digital phantoms were uniformly compressed numerically in the z-direction to a depth of 5 cm to simulate the compression that occurred when using our handheld reflection-mode DOT probe.^48^ Details of the simulated lesion and background tissue optical properties are in Table 1. For both simulation settings, the probe locations were moved slightly around the center locations to mimic a clinical scenario in which the probe is moved around the lesion location to obtain multiple sets of measurements from a single lesion. Then all measurements obtained from a single lesion were merged into one DOT histogram.

**Table 1:** Simulated lesion and background tissue optical properties in the pre-training dataset.

Target radius (cm)	Target center depth (cm)	Target $\mu_{a}$ (${\mathrm{cm}}^{-1}$)	Target ${\mu_{s}}^{\prime }$ (${\mathrm{cm}}^{-1}$)	Background tissue $\mu_{a}$ (${\mathrm{cm}}^{-1}$)	Background tissue ${\mu_{s}}^{\prime }$ (${\mathrm{cm}}^{-1}$)
0.375∼1.5	0.7∼2.7	0.06∼0.30	4∼8	FEM: 0.01∼0.06	FEM: 4∼8
				MC: 20 digital breast phantoms with fat fractions 20%∼80%^48^^,^^49^	

#### 2.1.1 US images

**Fig. 1:**
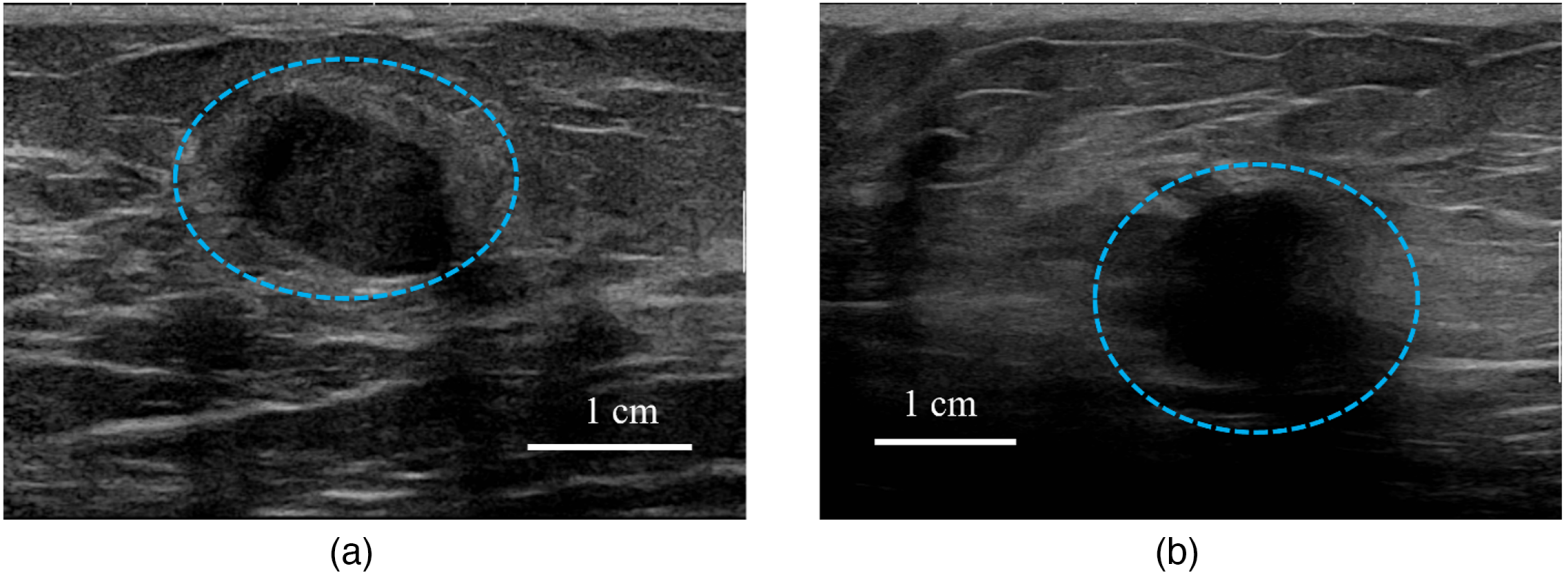
Examples of co-registered US images, with lesions marked by blue circles. (a) A benign lesion and (b) a malignant lesion.

Figure 1 shows co-registered US images of a benign lesion and a malignant lesion. The benign lesion has a clear boundary, whereas the malignant one has an irregular and ill-defined boundary. These features were used by the CNN to classify different types of breast lesions. To utilize CNN weights pre-trained with the ImageNet dataset,^50^ we cropped the lesion area of each US image to a region of interest (ROI) and resized it to 3×224×224  pixels before inputting it to the CNN. Here, 3 is the number of channels and 224×224 is the height × width of each channel. After resizing, the pixel sizes ranged from 0.044 to 0.123 mm. Finally, the images (ROIs) were normalized based on the mean and standard deviation of the ImageNet dataset.

#### 2.1.2 DOT frequency domain data (DOT histogram)

We normalized the measured frequency-domain diffuse reflectance obtained from the lesion side breast, $U_{l}$, to the contralateral normal breast, $U_{r}$, to produce the “perturbation”

$$\text{pert}=\frac{U_{l}-U_{r}}{U_{r}}=\frac{U_{l}}{U_{r}}-1=\text{real}(\text{pert})+j\times \mathrm{imag}(\text{pert}).$$

**Fig. 2:**
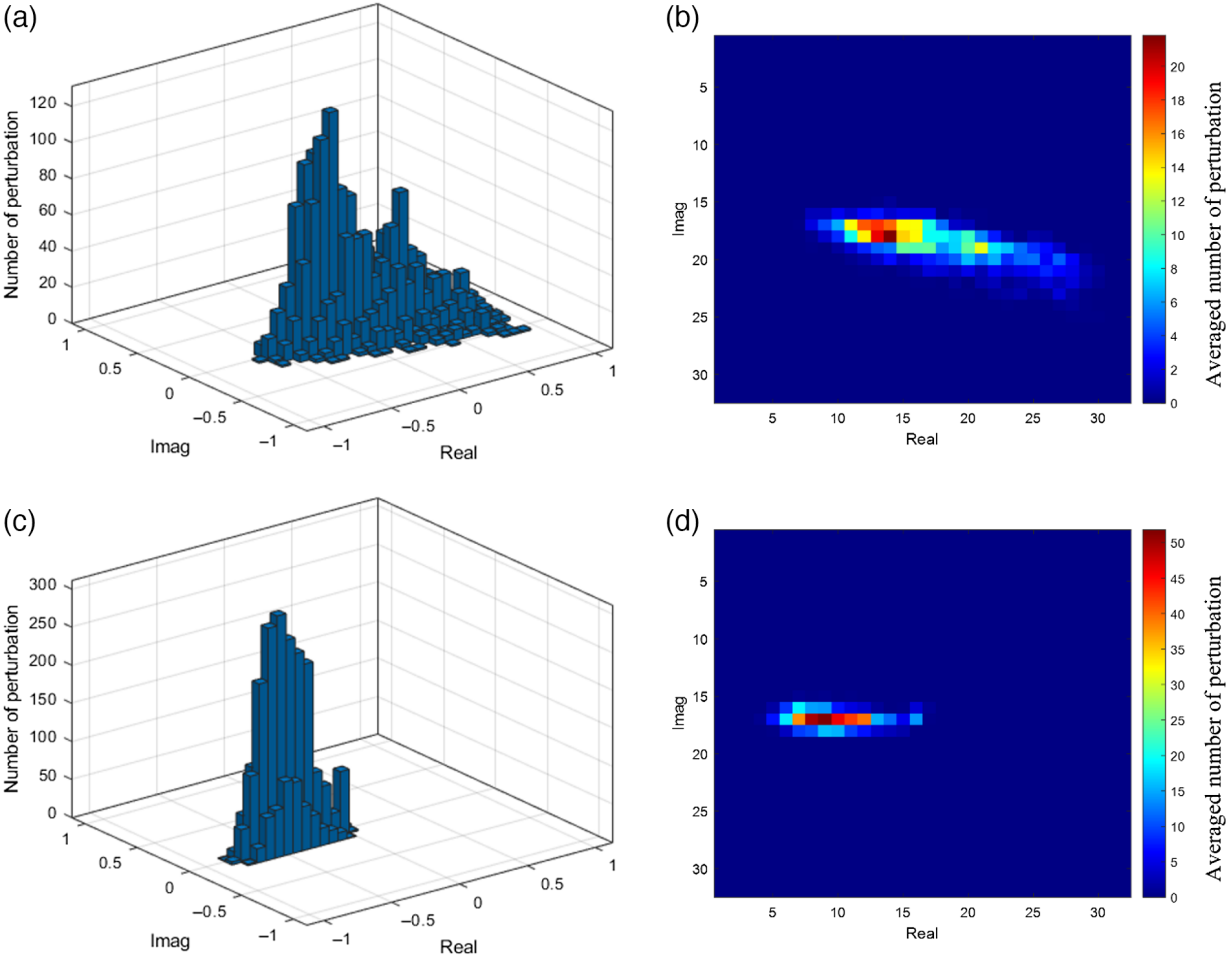
Examples of DOT histograms and 2D representations. (a) A benign case and (c) its 2D representation after averaging. (b) A malignant case and (d) its 2D representation after averaging.

We used a two-dimensional representation of the DOT perturbation measurements, with the x-axis being the real part of the perturbation and the y-axis being the imaginary part. For each lesion, the real and imaginary perturbation parts were used together to obtain a bivariate histogram with 32×32 bins, as shown in Fig. 2. For a benign lesion, which typically has lower absorption, the difference between the lesion side and reference side measurements is typically small, which means $U_{l}$ and $U_{r}$ are similar and their ratio is close to 1, so the points are more distributed around the origin. For a malignant lesion, typically with higher absorption, $U_{l}$ is smaller than $U_{r}$, so the perturbation is skewed toward −1 in the negative real axis. This histogram representation can handle different source-detector geometries. For each patient, all DOT perturbations from multi-spectral measurements and repeated lesion measurements were combined into one 2D histogram, and the total number of points in one 2D histogram is the number of sources × number of detectors × number of wavelengths × number of repeated lesion measurements. The histograms were then averaged based on the number of lesion files used, the number of wavelengths, and the number of detectors in the DOT system.

#### 2.1.3 DOT reconstructed images

**Fig. 3:**
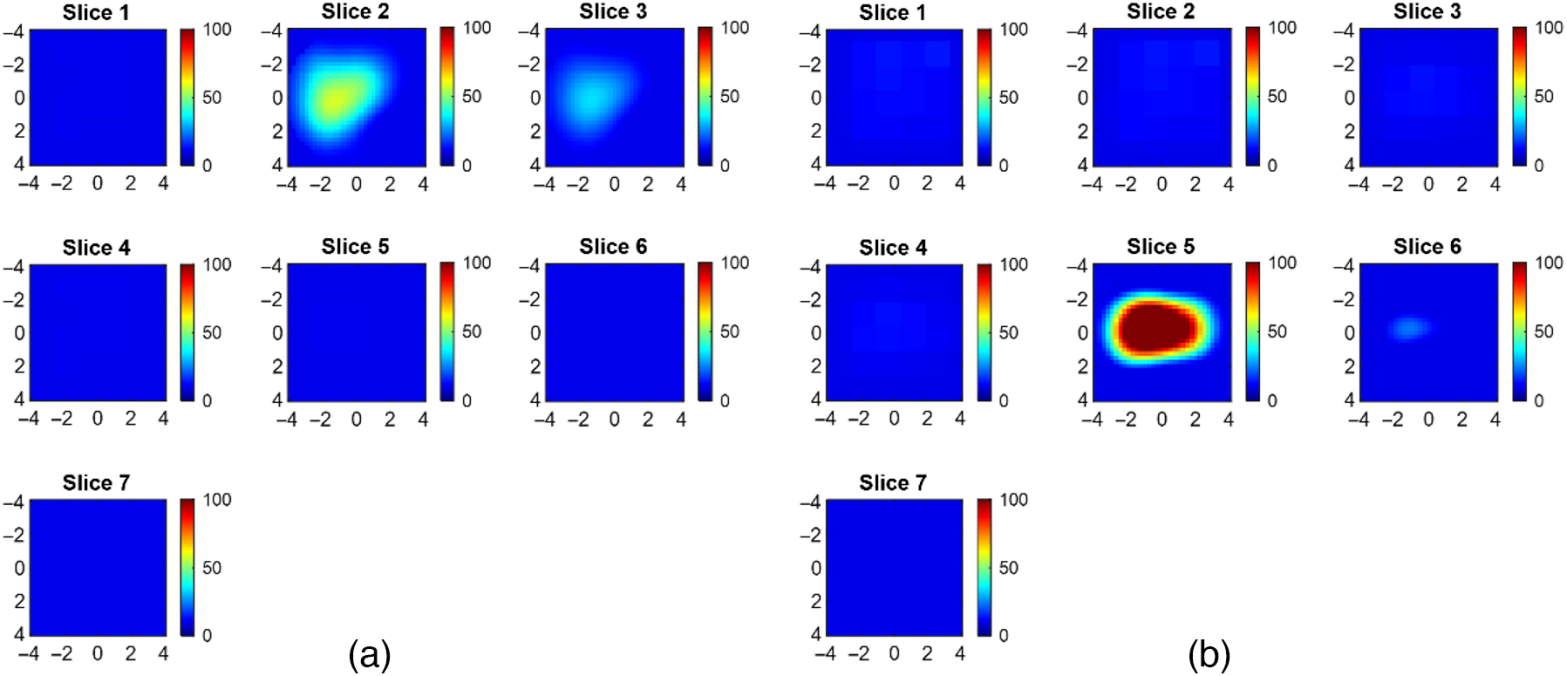
Examples of DOT reconstructed tHb images. Color bar indicates tHb values in μM. (a) A benign lesion and (b) a malignant lesion.

To reconstruct the unknown changes in the target absorption coefficients $\delta \mu_{a}$ compared to the reference side, we used the Born approximation to linearize the inverse problem:^51^

$$f(x)=\arg\;\min_{\delta \mu_{a}}\;({\left\|\mathrm{pert}-W\delta \mu_{a}\right\|}^{2}+\frac{\lambda }{2}{\left\|\delta \mu_{a}\right\|}^{2}),$$

where W is a sensitivity matrix calculated using the absorption and reduced scattering coefficients of the reference side and λ is a regularization parameter. The conjugate gradient algorithm and a dual mesh scheme using co-registered US guidance were employed to solve the inverse problem. After reconstructing the absorption maps, the total hemoglobin (tHb) distribution was calculated using all four wavelengths. Figure 3 shows reconstructed images of a benign and a malignant lesion, the same cases as in Fig. 2. The benign lesion has a relatively lower tHb concentration than the malignant lesion. Besides, the malignant lesion shows a “light shadowing” effect, because the tHb in the topmost layer is greater than the tHb of the underlying layers.^52^ The resulting high absorption by the cancer in the topmost layer significantly reduces the number of photons reaching the deeper layers.

### 2.2 First Stage

**Fig. 4:**
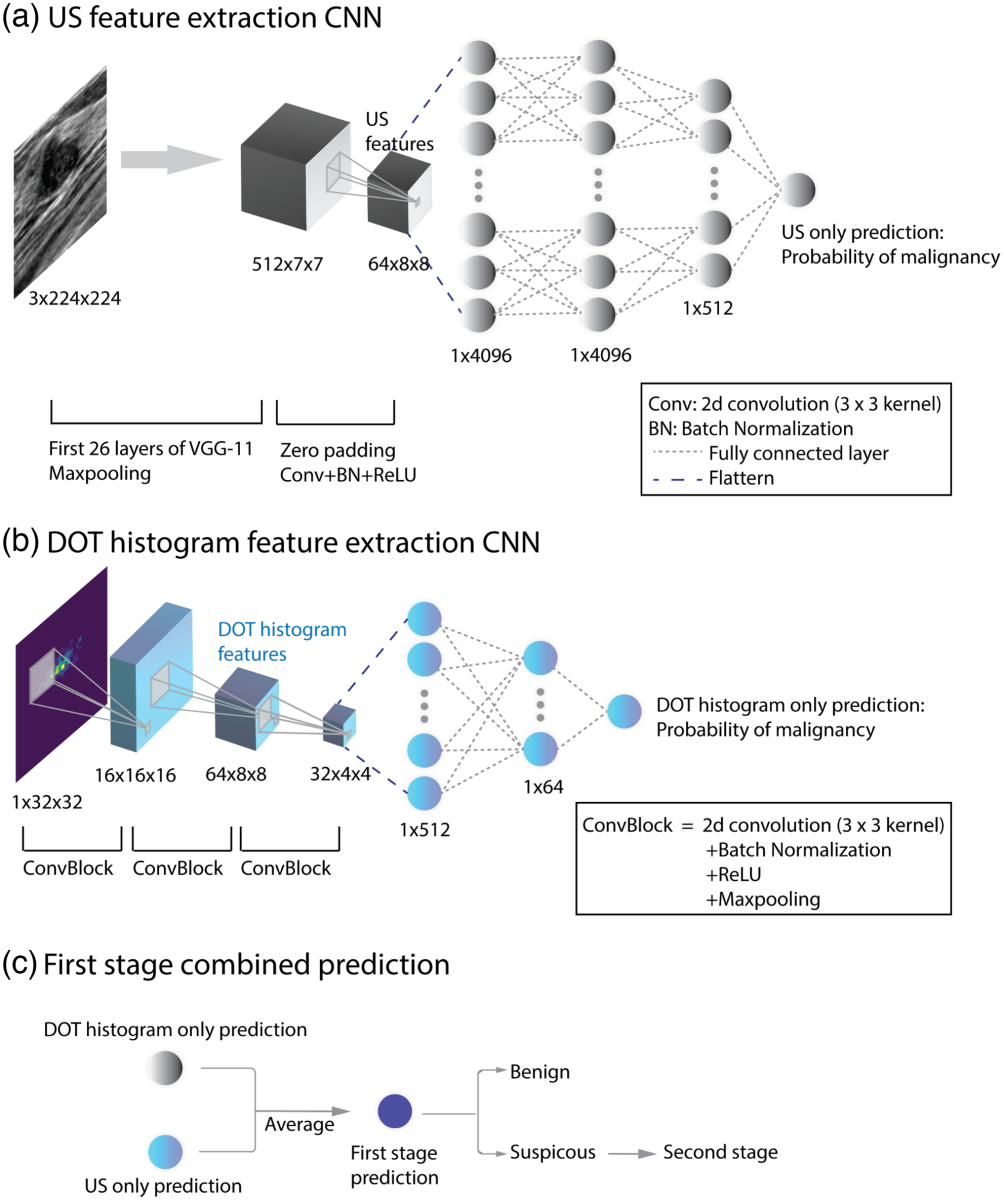
(a)–(c) First stage of the two-stage classification strategy.

In the first stage, two individual CNNs were trained with US images only and DOT histograms only, as shown in Figs. 4(a) and 4(b). Because we had a clear set of benign or malignant labels, supervised learning was used to train and evaluate all the CNN models.

#### 2.2.1 US only CNN

We used a modified VGG-11 neural network^53^ to extract features from co-registered US images, as shown in Fig. 4(a). The model was pre-trained with the ImageNet database. After the first 26 layers of the VGG-11 and a maxpooling layer, the output 512×7×7 matrix was zero-padded to a 512×8×8 matrix. Then another convolutional layer with a 3×3 kernel size and batch normalization was added to generate a 64×8×8 feature matrix. Following feature extraction, a binary classification model (benign versus malignant) was obtained using three fully connected layers. The weights of the first nine layers were kept unchanged from the pre-trained VGG-11 using ImageNet and then the model was fine-tuned using open-source US images^54^^,^^55^ and US images acquired with our US-guided DOT system. The fine-tuning was performed for 5 epochs with a learning rate of 0.001, followed by another 5 epochs with a learning rate of $1e^{-4}$, using stochastic gradient descent with a momentum of 0.9 as the optimizer. After training, the predicted probability of malignancy was extracted and combined with DOT histogram predictions for the first stage classification. The features before the fully connected layers were also extracted to be combined with DOT histogram features and DOT image features for the second stage classification.

#### 2.2.2 DOT histogram feature extraction CNN

The DOT histogram feature extraction CNN is shown in Fig. 4(b). The inputs are the 2D DOT histograms, measuring 1×32×32  pixels. After two convolutional layers with a 3×3 kernel size, followed by batch normalization and 2×2 maxpooling, the input histogram goes through another convolutional layer with a 3×3 kernel size and two fully connected layers. The output is the final prediction of the lesion type. We pre-trained the model with simulated DOT histograms for 20 epochs with a learning rate of ${1e}^{-4}$ and then fine-tuned it with patient data for 20 epochs with a learning rate of ${1e}^{-4}$. The Adam optimizer and the cosine annealing learning rate scheduler were used. After the model was trained, the predicted probability of benign versus suspicious was extracted and combined with US only predictions for first stage classification. After two convolutional layers, the features were extracted to be combined with US and DOT images in the second stage for identifying suspicious lesions.

#### 2.2.3 First stage combined prediction

In the first stage, as shown in Fig. 4(c), the probabilities of malignancy predicted from the US-only CNN and the DOT histogram-only CNN were averaged to generate the prediction of benign versus suspicious for near real-time diagnosis.

### 2.3 Second Stage

#### 2.3.1 DOT image feature extraction CNN

**Fig. 5:**
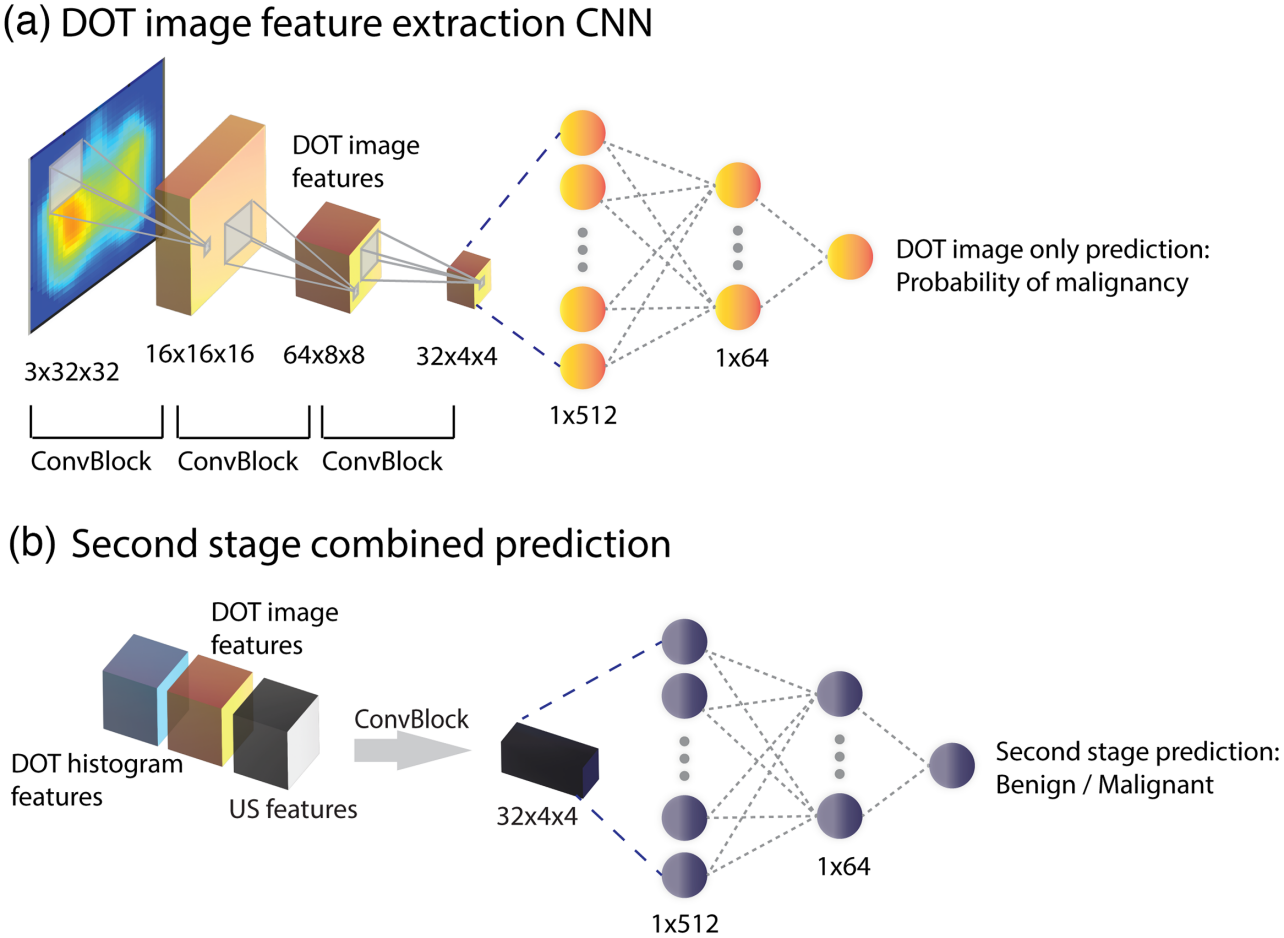
(a) and (b) Second stage of the two-stage classification strategy.

A similar CNN, as shown in Fig. 5(a), was built to extract DOT images. The inputs are the reconstructed tHb maps, which are 3×32×32 matrixes, and the output is the probability of the lesion being malignant. The three convolutional layers have a convolutional kernel of 3×3 and are followed by batch normalization, and two fully connected layers are applied to perform classification. The DOT image feature extraction CNN was trained on the simulation data with a learning rate of 0.001 for 60 epochs and then fine-tuned with patient data with a learning rate of 0.001 for 10 epochs. The features after two convolutional layers were extracted to be combined with DOT histogram features and US features for the second-stage classification.

#### 2.3.2 Two-stage Classification Strategy

As shown in Fig. 5(b), in the second stage, DOT image features, DOT histogram features, and US features are concatenated to generate 192×8×8 input matrices, followed by a convolutional layer with a 3×3 kernel size and two fully connected layers to output the final prediction of the lesion type for the suspicious group from the first stage. In the two-stage strategy, to recover the optical properties of the suspicious lesions, DOT reconstruction is performed for the suspicious cases identified by the first stage classifier. Then, the DOT image features are extracted using the DOT image feature extraction CNN. Next, to obtain the final classification, the DOT histograms, DOT image features, and US features are concatenated and fed into the second stage classifier. The final predictions for all cases thus comprise the benign predictions from the first stage and the predictions of suspicious cases from the second stage.

**Fig. 6:**
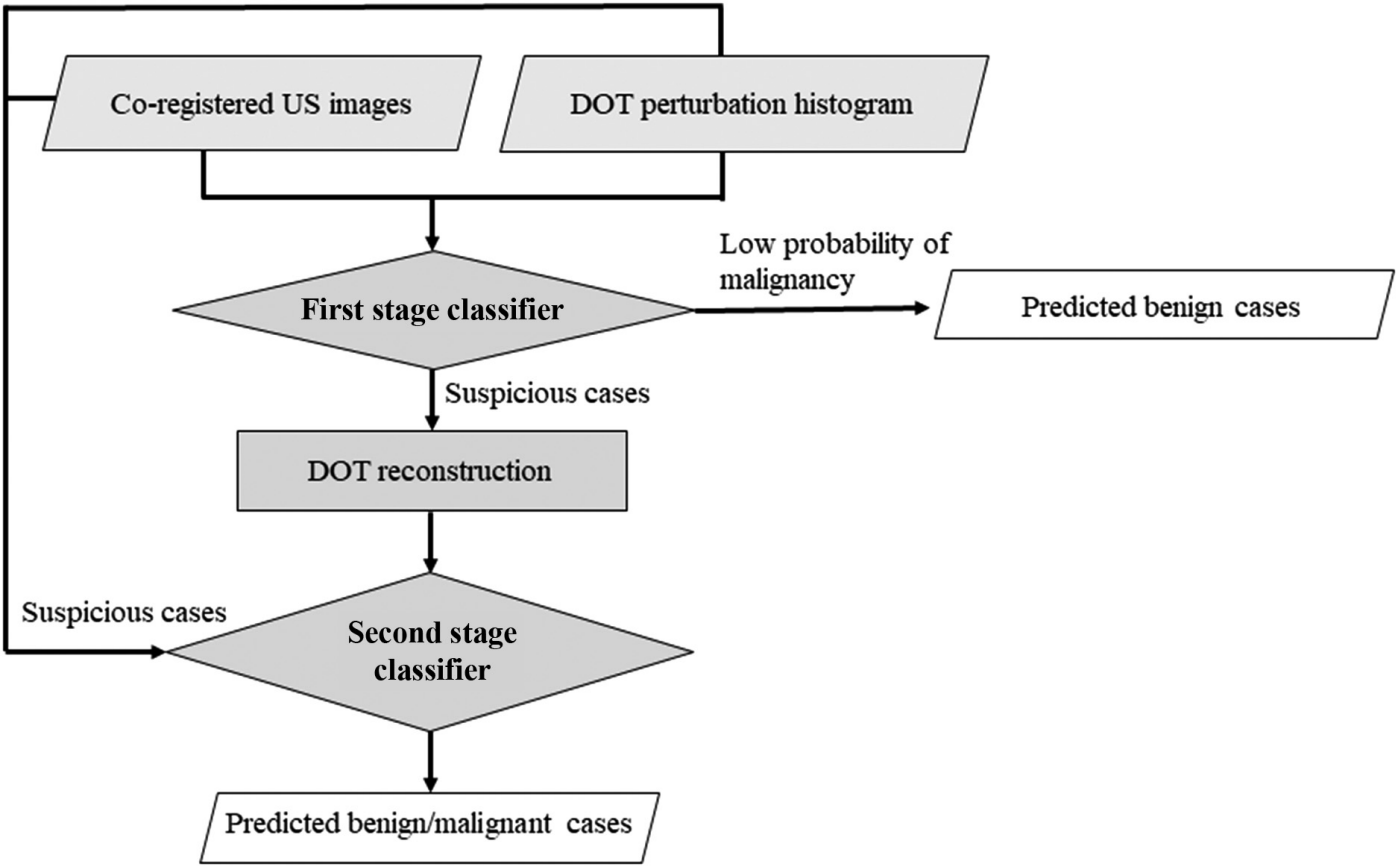
Two-stage classification workflow.

The two-stage strategy is shown in Fig. 6. Once the data acquistion is complete, the co-registered US images and the perturbation measurements are generated and put into the first stage classifier, which combines the predictions from the US only CNN and DOT histogram only CNN. To lower the false negative rate while maintaining a high true negative rate, the prediction threshold is set at a value lower than 0.5, the value commonly used in binary classification. The threshold for the first stage classification model was determined by a grid search of all thresholds lower than 0.5, with a step size of 0.01. Based on the grid search results, we set a threshold value of 0.12, which minimized the false negative rate while maintaining high accuracy in predicting benign cases. Lesions with predicted probabilities lower than the threshold are classified as benign lesions, and the rest are classified as suspicious lesions.

## 3 Experimental Results

The Supplementary Material provides the number of trainable parameters for each model as well as examples of training and testing losses.

### 3.1 First Stage Classification Results

On average, the first stage identified 18.2 of 25 cases as benign, so the benign accuracy was 73.0%. However, an average of 0.7 malignant cases were falsely classified as benign, resulting in a false negative rate of 3.0%. Among the 50 testing set cases, an average of 30.1 cases were classified into the suspicious group and fed into the second stage classifier. These results are better than those from US-only models, which can filter out only 68.1% benign cases, with a false negative rate of 9.8%, or histogram-only models, which can filter out 54.6% benign cases, with a false negative rate of 9.4%.

### 3.2 Two-Stage Classification Strategy Results

**Fig. 7:**
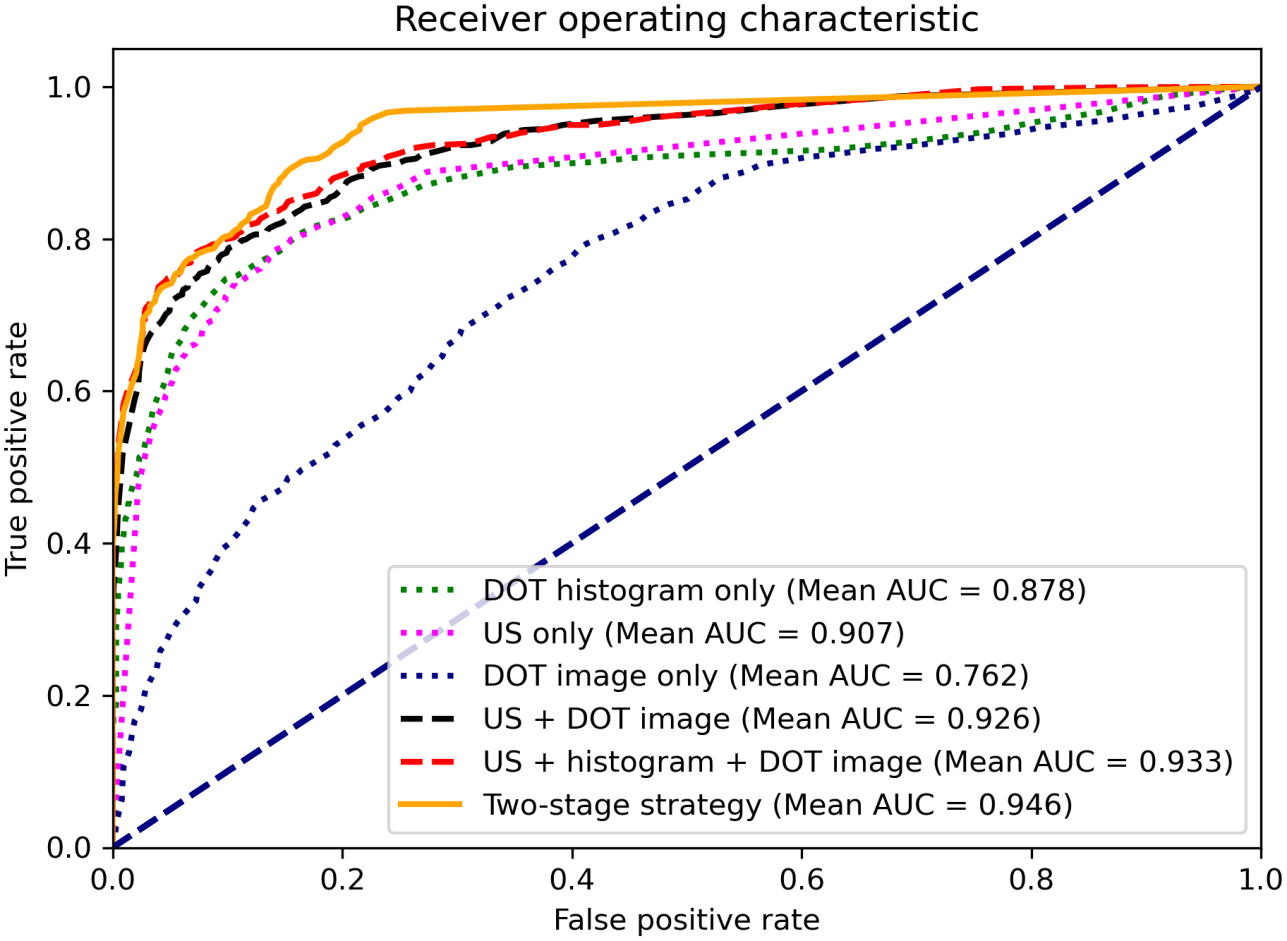
ROC curves for testing data.

After the first stage classifier excluded part of the benign cases, the suspicious cases above the threshold, with a higher probability of malignancy, were advanced into the second stage classifier. For those cases, DOT images were reconstructed using the conjugate gradient descent method with regularization, and the reconstructed image features were extracted using the DOT image feature extraction CNN. Next, the reconstructed DOT image features, DOT histogram features, and US features for the suspicious group were put into the second stage classifier to obtain the final classification. The averaged ROCs from 50 runs for the two-stage classification strategy, along with the single-modality models, are shown in Fig. 7. The first stage classifier predicts only two groups: benign and suspicious cases. In the second stage, the suspicious cases are further classified as either benign or malignant. Biopsy confirmed the true positive and false positive predictions from the second stage, and the biopsy-confirmed true negative and false negative predictions from both stages were used to generate the two-stage combined ROC curve. The confusion matrix used to generate the ROC curve is shown in Table 2, where a 1 or 2 denotes results from the first or second stage. TM, FM, FB, and TB respectively denote true malignant, false malignant, false benign, and true benign. As shown in Fig. 7, the two-stage classification strategy provides a more accurate classification (AUC=0.946, 95% CI: 0.939 to 0.954) than any of the single-modality based classifiers.

**Table 2:** Confusion matrix of two-stage combined results, where a 1 or 2 denotes results from the first or second stage. TM, FM, FB, and TB denote true malignant, false malignant, false benign, and true benign, respectively.

Predicted	Biopsy	
	Malignant	Benign
**Malignant**	TM2	FM2
**Benign**	FB2 + FB1	TB2 + TB1

We also evaluated the performance of a single-stage model that combines features extracted from DOT histograms, DOT images, and US images. For a fair comparison, the evaluation is performed for all testing cases, not limited to only the suspicious cases classified by the first stage model. This model achieved an AUC of 0.933 (95% CI: 0.924 to 0.941), which is compariable with that of the two-stage classification strategy; however, the latter can achieve near real time diagnosis for 70% of the benign lesions.

### 3.3 Comparison with Traditional Feature-Based Machine Learning Approaches

As a comparison, we manually extracted features from DOT histograms and DOT images following Ref. 28 and used two radiologists’ BI-RADS readings. Then we used random forest or SVM classifiers instead of deep learning classifiers to perform the classification, with the results shown in Table 3.

**Table 3:** Comparison of deep-learning based classification with traditional feature-based classification.

Traditional ML input	Traditional ML method	Traditional ML Performance	Deep learning counterpart input	Deep learning counterpart performance
12 DOT histogram features	Random forest classifier	AUC = 0.715	DOT histograms	AUC = 0.878
First stage: 12 DOT histogram features and BI-RADS from 2 radiologists	Random forest classifier	61.8% of benign cases were singled out, with a 6.6% false negative rate	First stage: DOT histograms and US images	73.0% of benign cases were singled out, with a 3.0% false negative rate
2 DOT functional features: total hemoglobin and oxy hemoglobin	SVM classifier	AUC = 0.744	DOT reconstructed total hemoglobin maps	AUC = 0.762
DOT histogram features, BI-RADS, and DOT functional features	Random forest classifier	AUC = 0.931	DOT histograms, US images, and DOT reconstructed total hemoglobin maps	AUC = 0.933
Two-stage classification strategy	First stage: DOT histogram features and BI-RADS	AUC = 0.925	Two-stage classification strategy	AUC = 0.946
	Second stage: DOT histogram features, BI-RADS, and functional features			

These findings suggest that deep learning methods outperform traditional ML methods when using DOT histograms and DOT images only. Additionally, deep learning models combining DOT with US features achieve results better than or comparable to the results of traditional ML models utilizing radiologists’ BI-RADS readings.

## 4 Conclusion

In this paper, we proposed a two-stage strategy using deep-learning models to classify whether a breast lesion is malignant or benign. In the first stage, DOT histogram features and US features are extracted using two feature extraction CNN models. Next, to single out those benign cases with a low probability of malignancy, the averaged prediction scores from the two models are calculated as the first stage result, which yields predictions of benign or suspicious. This strategy can identify 73.0% of benign cases in the first stage without the need for imaging reconstruction. In the second stage, the DOT image features, extracted by another feature extraction CNN model, are combined with the DOT histograms and US features and input into the second-stage classifier to obtain the final diagnostic classification. This strategy achieved near real-time diagnosis for a significant portion of breast lesions, without the need for image reconstruction in the first stage, and the second stage ensures the overall prediction accuracy. Combining the first stage’s high sensitivity and the second stage’s high specificity, the two-step classification strategy provides better classification accuracy, with an AUC of 0.946, than either single-modality-only models or a single-stage classification model that combines all features.

The proposed two-stage classification strategy has shown promising results on clinical patient data, with high robustness over 50 random training-testing splits. However, there are still limitations to this approach. First, the first-stage model has a false negative rate of 3.0%, even with a threshold set much lower than 0.5. This performance could be improved by incorporating radiologists’ BI-RADS scores, which have extremely high sensitivity, albeit low specificity. Second, the second-stage model involves DOT image reconstruction, which requires manual data preprocessing, such as reference selection and outlier removal. This limitation can be mitigated by applying an automatic data preprocessing strategy to fully automate the entire two-stage classification strategy. Additionally, we utilized traditional iterative methods for DOT reconstruction to accommodate data collected from various DOT systems, and these methods are usually slower than deep learning reconstruction. As more data are accumulated from a specific DOT system, deep learning reconstruction^37^ could be used to achieve real-time diagnosis for all cases. Furthermore, with more data in the future, it will be possible to reserve an additional testing set that has never been exposed to the model during training and thus the robustness of the model could be further evaluated.

## Code, Data, and Materials Availability

Associated code has been uploaded to https://github.com/OpticalUltrasoundImaging/DOT_two_stage. Data are available from the corresponding author upon reasonable request.
