# Supplementary material for: Two-stage classification strategy for breast cancer diagnosis using ultrasound-guided diffuse optical tomography and deep learning
Source: J Biomed Opt. 2023 Aug 26;28(8):086002. doi: 10.1117/1.JBO.28.8.086002 (PMC10457211; doi:10.1117/1.JBO.28.8.086002)
Supplement: Supplementary file 1 [file JBO_028_086002_SD001.docx]

Table S1. Trainable parameters in CNNs

| **CNN** | **US image only** | **DOT histogram only** | **DOT image only** | | **DOT image features + US features** | | **DOT histogram features + US features + DOT image features** | |
| --- | --- | --- | --- | --- | --- | --- | --- | --- |
| **# of parameters** | 25669314 | 44593 | 61313 | 69857 | | 88289 | |  |

**
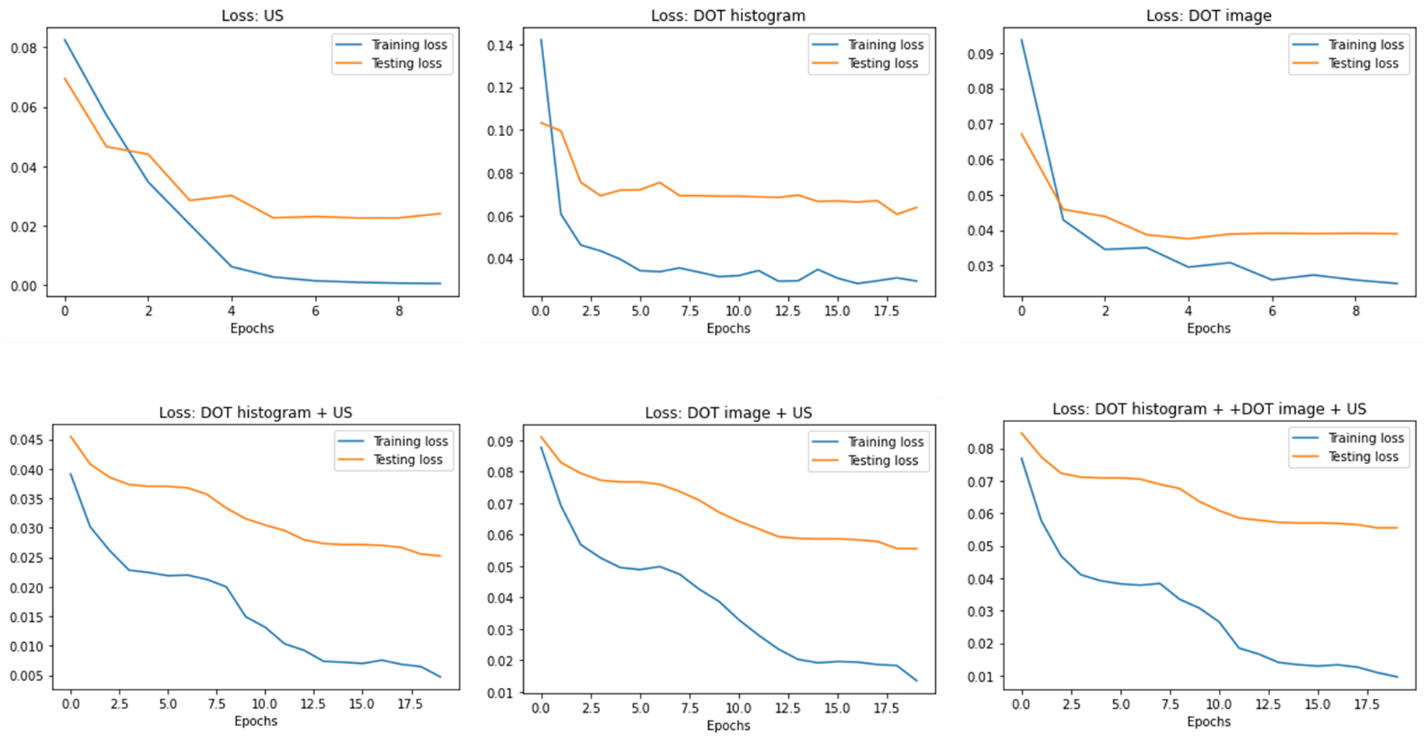
**

**Figure S1.** Examples of training and testing losses
